# Cost of hepatic decompensation and liver transplantation events in primary biliary cholangitis: a retrospective observational study

**DOI:** 10.57264/cer-2025-0110

**Published:** 2026-02-16

**Authors:** Robert G Gish, Joanna P MacEwan, Yutong Liu, Dannielle Lebovitch, Radhika Nair, Leona Bessonova, Jing Li, Zobair M Younossi

**Affiliations:** 1 Robert G. Gish Consultants, LLC, San Diego, CA 92037, USA; 2 Genesis Research Group, Hoboken, NJ 07030, USA; 3 Intercept Pharmaceuticals, Inc., Morristown, NJ 07960, USA; 4 Beatty Liver and Obesity Research Program, Inova Health System, Falls Church, VA 22042, USA; 5 Center for Outcomes Research in Liver Disease, Washington, DC 20037, USA

**Keywords:** Claims, cost analysis, healthcare resource utilization, hepatic decompensation, hospitalization, liver disease, liver transplantation, observational study, primary biliary cholangitis, real-world evidence

## Abstract

**Aim:**

Complications of primary biliary cholangitis (PBC) are proposed to confer substantial economic burden to patients and healthcare systems. This retrospective observational study evaluated the cost of PBC-related hepatic decompensation and liver transplantation using a large administrative claims database.

**Materials & methods:**

Patients aged ≥18 years at the time of first claim with a diagnosis of PBC were identified using Optum’s de-identified Clinformatics^®^ Data Mart Database. Two cohorts were established based on the type of event (hepatic decompensation or liver transplantation) that patients experienced on or after the date of their first claim with the PBC diagnosis. Costs for the hepatic decompensation hospitalization and 30-day post-discharge period were examined at the event level. Hospitalizations occurring within the 30-day post-discharge period after a hepatic decompensation event were considered readmissions, and costs from the initial event were combined with those from the ensuing readmissions. In the liver transplantation cohort, costs for the pretransplant evaluation, hospitalization for transplantation, and post-transplant care and complications were assessed per patient.

**Results:**

A total of 2118 and 163 patients met study inclusion criteria in the hepatic decompensation and liver transplantation cohorts, respectively. The overall mean cost per hepatic decompensation event (n = 3581) was $63,612.09. The mean cost per event with readmission within 30 days (n = 991, 27.7%) was $116,424.25; for events without readmission, the mean cost was $43,404.81. The mean total cost of liver transplantation per patient was $328,336.60. The mean cost per patient was highest for the hospitalization for transplantation ($226,908.70).

**Conclusion:**

This comprehensive cost analysis demonstrates the high-cost burden of PBC disease progression. Appropriate patient management may help to mitigate the economic burden of PBC-related hepatic decompensation and liver transplantation.

## Plain language summary: Cost of liver dysfunction/decompensation & transplantation in patients with primary biliary cholangitis

**What is this article about?:**

Primary biliary cholangitis (PBC) is a chronic liver disease that will damage the liver over time, potentially leading to liver dysfunction (also called hepatic decompensation), liver failure requiring transplantation and death. This study evaluated the costs of PBC-related hepatic decompensation and liver transplantation in adult patients with PBC using a large national insurance claims database.

**What methodology is described?:**

Costs for hepatic decompensation included costs from the hospitalization and 30-day period after discharge from the hospital. Costs for liver transplantation included a pretransplant evaluation, the hospitalization period and 1 year of post-transplant care, including the management of potential complications.

**What were the results?:**

The average cost of a hospital stay for hepatic decompensation was $63,612.09. The average cost of a hospital stay was $116,424.25 when patients were readmitted to the hospital within 30 days after discharge (hospitalization costs for both stays combined). The average cost of liver transplantation per patient was $328,336.60. The hospitalization for transplantation was more expensive than the pretransplant evaluation or 1 year of post-transplant care.

**What do the results mean?:**

The findings highlight the high cost burden associated with hepatic decompensation and liver transplantation. Use of current and emerging PBC therapies can potentially help reduce the risk for these events and avoid future associated costs.

## Shareable abstract

This retrospective observational study using a large national claims database demonstrates that #primarybiliarycholangitis-related hepatic decompensation and liver transplantation are costly complications of disease progression for #patients and #healthcare systems

Primary biliary cholangitis (PBC) is a chronic cholestatic liver disease characterized by autoimmune destruction of the small intrahepatic bile ducts [1,2]. PBC is more common among women and is typically diagnosed between 40 and 60 years of age [1,2]. The prevalence of PBC has increased over time, possibly due to increased testing or changes in test sensitivity, with a recent US estimate of 40.9 per 100,000 adults [3,4]. Although disease progression is typically slow, when left untreated, PBC will lead to late-stage disease progression in most patients, including hepatic decompensation, liver failure requiring transplantation and/or death [1,2,5]. Therefore, the goals of treatment are to prevent disease progression and its associated complications [2,5–7].

The first-line treatment for PBC is ursodeoxycholic acid (UDCA), which has a demonstrated long-term benefit on transplant-free survival [7,8]. Ocaliva^®^ (obeticholic acid) has been evaluated for second-line treatment of PBC [9–13] but was voluntarily withdrawn from the US in September 2025 following the US FDA’s request related to results following Ocaliva’s^®^ confirmatory study [13,14]. Elafibranor and seladelpar received accelerated approval in 2024 for the second-line treatment of patients with PBC who have inadequate response or intolerance to UDCA based on reductions in alkaline phosphatase levels [15,16]. Potential third-line treatments include fenofibrate and bezafibrate, neither of which have been formally approved from a regulatory perspective, and there are no phase III trials to support their use in PBC.

Chronic, progressive diseases such as PBC confer substantial economic burden to patients and healthcare systems [17,18]. PBC is associated with rising and considerable healthcare resource utilization (HCRU), including hospitalizations for disease complications [19–21]. Inpatient stays, in particular, comprise a large proportion of costs associated with PBC [20,21]. Patients whose disease has progressed to cirrhosis and its associated complications, such as portal hypertension, ascites and hepatic encephalopathy, also experience higher HCRU compared with patients without cirrhosis [19–22]. Furthermore, hospitalizations and emergency department visits are more common in patients with cirrhosis than in patients without cirrhosis [19,22].

The economic burden of PBC-related complications of disease progression, specifically hepatic decompensation at an event level, has not been examined. While the cost of liver transplantation has been assessed in patients with other liver diseases such as nonalcoholic steatohepatitis [23], it has not been comprehensively estimated in patients with PBC. The objectives of this study were to estimate the real-world cost of hospitalization for hepatic decompensation (including readmissions) at an event level and the cost of liver transplantation in patients with PBC using a large administrative claims database.

## Materials & methods

### Study design & data source

This retrospective observational study was conducted using Optum’s de-identified Clinformatics^®^ Data Mart (Optum^®^ CDM) Database, which contains de-identified inpatient, outpatient and pharmacy claims from patients in the US. Optum CDM contains claims data from 81 million patients with commercial or Medicare insurance coverage across all US states [24]. Claims data refer to administrative health insurance records that are collected through health insurance claims for billing and reimbursement of services and procedures rendered [25].

### Study population

Patients with a diagnosis of PBC based on the International Classification of Diseases (ICD)-10-Clinical Modification code K74.3 in any position (e.g., primary or secondary) in ≥1 inpatient claim or ≥2 outpatient claims on separate days were identified in Optum CDM between 1 January 2016 and 14 November 2023 (study identification period). The use of ICD-10-Clinical Modification code K74.3 was based on previous studies that have validated the use of ICD codes to identify patients with PBC from administrative data [26,27]. Patients must have been aged ≥18 years at the time of first claim with the diagnosis of PBC. While this first claim with the diagnosis of PBC must have occurred during the study identification period, patients may have been originally diagnosed with PBC before the start of the study period.

**Figure 1.**
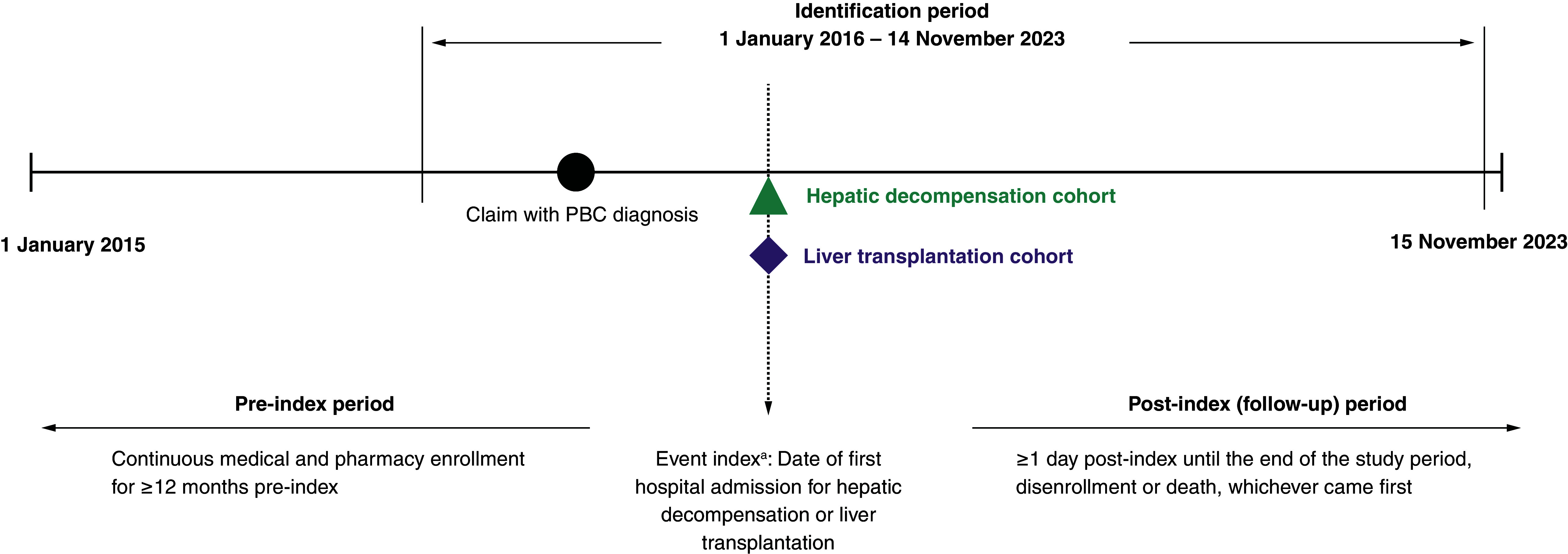
Study design. ^a^The event index for each cohort was set as the date of first hospital admission for hepatic decompensation or liver transplantation that occurred on or after the first claim with the PBC diagnosis. PBC: Primary biliary cholangitis.

Two cohorts were established based on the type of event (hepatic decompensation or liver transplantation) that patients experienced on or after the first claim with the diagnosis of PBC. The event index for each cohort was set as the date of first hospital admission for hepatic decompensation or liver transplantation that occurred on or after the first claim with the PBC diagnosis (Figure 1). Patients were required to have continuous medical and pharmacy enrollment for ≥12 months pre-index and ≥1 day post-index. Patients were followed from the event index until the end of the study period (15 November 2023), disenrollment or death, whichever came first.

### Patient characteristics

Patient characteristics, including age, sex, race/ethnicity, type of insurance coverage (Medicare versus commercial), follow-up time and use of UDCA, were assessed at the patient level during the 12-month pre-index period. Patients’ pre-index comorbidity profiles using the Charlson Comorbidity Index (CCI) were also assessed [28], along with the proportion of patients diagnosed with concomitant autoimmune diseases.

### Hepatic decompensation cohort

In the hepatic decompensation cohort, costs for the hospitalization period and the 30-day post-discharge period were evaluated. Hepatic decompensation events were defined as any hospitalization with diagnosis codes for a decompensation-related condition or procedure [22,29,30]. Decompensation-related diagnoses included ascites, spontaneous bacterial peritonitis, jaundice, esophageal or gastric varices with bleeding, hepatic encephalopathy and/or portal hypertension (a full list of hepatic decompensation-related diagnosis codes and descriptions is provided in Supplementary Table 1). Decompensation-related procedures included paracentesis, transjugular intrahepatic portosystemic shunt (TIPS) and/or esophageal or gastric varices treatment (ligation, banding, tamponade or sclerotherapy; a full list of hepatic decompensation-related procedure codes and descriptions is provided in Supplementary Table 2). Given the specificity of these complications of hepatic decompensation, a formal diagnosis of cirrhosis was not required.

All events of hepatic decompensation during the study identification period were recorded for each patient, and events were grouped together when occurring within a short time frame. Rehospitalizations occurring within the 30-day post-discharge period after a previous event were considered as readmissions. The initial event and any subsequent readmissions were grouped and analyzed as part of the same event. Each event spanned from the admission date of the first decompensation event to the last discharge date.

During the post-discharge period, certain medications, laboratory assessments, procedures and physician visits were examined as part of the costs of the hepatic decompensation event, based on guidance from hepatologists and 2021 AASLD guidelines [31]. Use of rifaximin, lactulose, lactitol, beta-blockers (entire class), spironolactone, furosemide, torsemide, bumetanide, antibiotics (only if administered within 30 days following a diagnosis of spontaneous bacterial peritonitis), octreotide and other somatostatin analogs, and terlipressin during the post-discharge period were included in the costs of the hepatic decompensation event. Certain laboratory assessments (comprehensive metabolic panel; complete blood count; liver panel, including liver enzymes [LEs] and liver function tests [LFTs; e.g., total bilirubin and albumin]; coagulation studies [prothrombin time, partial thromboplastin time, thrombin time and international normalized ratio]), procedures (paracentesis, TIPS and esophageal or gastric varices treatment) and radiologic imaging studies (computed tomography, MRI and abdominal ultrasound) occurring during the post-discharge period were also incorporated into the costs of the hepatic decompensation event. Additionally, all specialist visits (gastroenterology or hepatology; Supplementary Table 3) or outpatient visits to any specialty with claims including liver-related diagnosis codes during the post-discharge period were included in the costs of the hepatic decompensation event.

### Liver transplantation cohort

In the liver transplantation cohort, costs for the pretransplant evaluation, hospitalization for transplantation and post-transplant care and complications were evaluated. The pretransplant evaluation, per 2013 AASLD guidelines [32], included a gastroenterologist/hepatologist encounter, general health and dental assessment, mental health professional consultation, nutritional evaluation, cardiac evaluation, laboratory testing, infection screening and hepatic imaging performed within the 6 months before liver transplantation hospitalization (Supplementary Table 4). The hospitalization was defined as a hospitalization event with a liver transplantation procedure code (Current Procedural Terminology codes and descriptions for liver transplantation procedures are provided in Supplementary Table 5). Post-transplant care and complications included any inpatient, outpatient or emergency department encounter mentioning liver transplantation, transplant care, transplant complications (nonspecific complications and biliary complications [cholestasis, sepsis and bacterial cholangitis]), rejection or liver biopsy occurring during the 1 year following liver transplantation (beginning when the patient was discharged from the hospital).

During the same 1-year period, use of prednisone, methylprednisolone, cyclosporine, tacrolimus, azathioprine, 6-mercaptopurine, mycophenolate mofetil, mycophenolic acid, sirolimus and everolimus were considered related to post-transplant care. Certain laboratory assessments (comprehensive metabolic panel; complete blood count; liver panel, including LEs and LFTs; coagulation studies; and serum drug level measurements for immunosuppressants) were also deemed related to post-transplant care. Additionally, certain vaccinations (influenza, pneumonia, COVID-19, hepatitis A and B and varicella-zoster), hospitalizations with infections in the primary or secondary diagnostic position (suggesting that infection was the chief complaint on admission) and encounters with a gastroenterologist or hepatologist were considered related to post-transplant care (Supplementary Table 3).

### Statistical analyses

In the hepatic decompensation cohort, mean (SD), median (IQR) and total cost range were evaluated at the event level (i.e., hospital stay). Descriptive analyses were stratified by events with and without readmission within 30 days. For events with readmission(s), the time to first readmission within the event and the number of readmissions were evaluated. Additionally, for events with readmission(s), costs from the initial hepatic decompensation event were combined with those from the ensuing readmission events and analyzed as a single event. Length of hospital stay was also assessed and similarly stratified by events with and without readmission within 30 days.

Costs for liver transplantation were assessed for the pretransplant evaluation, hospitalization for transplantation and post-transplant care and complications. Mean (SD), median (IQR) and cost range were evaluated per patient.

This analysis was conducted from a payer perspective, and only direct medical costs were assessed in this cost analysis; indirect costs (e.g., productivity loss, caregiver burden and premature mortality) were not examined.

## Results

### Patient characteristics

**Figure 2.**
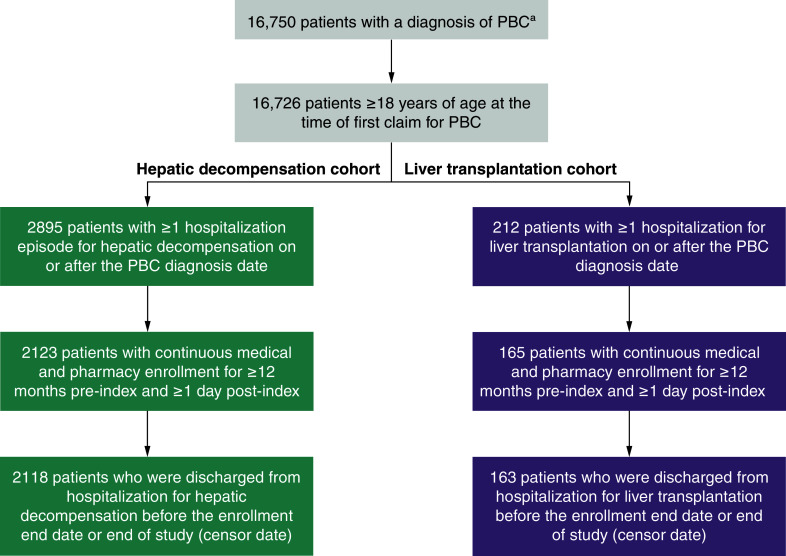
Attrition of patients in the hepatic decompensation and liver transplantation cohorts. ^a^Diagnosis of PBC was based on either ≥1 inpatient claim with a PBC admission diagnosis at any position (e.g., primary or secondary) or ≥2 outpatient claims with a PBC diagnosis on separate days. PBC: Primary biliary cholangitis.

A total of 16,726 patients met diagnostic criteria for PBC and were aged ≥18 years at the time of first claim for PBC (Figure 2). In the hepatic decompensation cohort, 2118 patients met all study inclusion criteria, and 163 patients met all study inclusion criteria in the liver transplantation cohort.

The mean (SD) age was 69.2 (12.4) and 55.5 (13.4) years in the hepatic decompensation and liver transplantation cohorts, respectively (Table 1). Most patients (70.7%) were female in the hepatic decompensation cohort, while approximately half (52.8%) were female in the liver transplantation cohort. In the hepatic decompensation cohort, Medicare was the most common insurance type (80.5%); commercial insurance was more common (57.1%) in patients undergoing liver transplantation. Only approximately half of patients in the hepatic decompensation and liver transplantation cohorts (51.0% and 54.6%, respectively) filled prescriptions for UDCA during the 12-month pre-index period. Nearly all patients (92.4% and 98.2% in the hepatic decompensation and liver transplantation cohorts, respectively) had a CCI >1.

**Table 1.** Patient characteristics: demographic and clinical.

Characteristic	Hepatic decompensation cohort (n = 2118)	Liver transplantation cohort (n = 163)
Pre-index^†^		
Age, mean (SD), years	69.2 (12.4)	55.5 (13.4)
Sex, female, n (%)	1497 (70.7)	86 (52.8)
Race/ethnicity, n (%)		
Asian	40 (1.9)	2 (1.2)
Black	216 (10.2)	13 (8.0)
Hispanic	337 (15.9)	22 (13.5)
White	1389 (65.6)	117 (71.8)
Unknown/missing	136 (6.4)	9 (5.5)
Type of insurance coverage, n (%)		
Commercial	409 (19.3)	93 (57.1)
Medicare	1706 (80.5)	69 (42.3)
Multiple	3 (0.1)	1 (0.6)
UDCA use, n (%)	1080 (51.0)	89 (54.6)
Concomitant autoimmune diagnosis, n (%)	526 (24.8)	46 (28.2)
CCI		
Mean (SD)	5.6 (3.0)	5.8 (2.5)
Median (IQR)	5.0 (3.0, 8.0)	6.0 (4.0, 7.0)
CCI >1, n (%)	1958 (92.4)	160 (98.2)
**Post-index**		
Follow-up time, days		
Mean (SD)	482.0 (580.0)	743.2 (624.3)
Median (IQR)	246.5 (74.0, 657.0)	561.0 (237.0, 1063.0)

† The pre-index period comprised the 12 months before the event index.

CCI: Charlson Comorbidity Index; IQR: Interquartile range; SD: Standard deviation; UDCA: Ursodeoxycholic acid.

### Hepatic decompensation

The 2118 patients in this hepatic decompensation cohort had 3581 hepatic decompensation events, indicating that some patients had >1 event. In this event-level analysis, the overall mean (SD) length of hospital stay was 14.16 (20.26) days (Table 2). The mean (SD) length of hospital stay for events with readmission within 30 days was 29.49 (30.72) days; the mean (SD) length of stay for events without readmission was 8.30 (9.06) days. A total of 991 of 3581 (27.7%) events of hepatic decompensation had ≥1 readmission within 30 days. Among the events with readmission within 30 days, the mean (SD) time to first readmission within the event was 8.35 (8.94) days. The mean (SD) number of readmissions was 1.78 (1.42), with a median (IQR) of 1.00 (1.00–2.00) and range of 1.00–12.00.

**Table 2.** Event-level analysis of hepatic decompensation cost, readmissions and length of hospital stay (n = 2118).

Cost per event, $	Overall (events, n = 3581)^†^	No readmission (events, n = 2590)	Readmission within 30 days (number of events = 991)
Mean (SD)	63,612.09 (83,281.84)	43,404.81 (50,566.20)	116,424.25 (120,557.91)
Median (IQR)	36,074.51 (22,637.66; 68,780.63)	29,728.97 (19,975.69; 40,929.44)	74,024.62 (53,870.12; 128,407.82)
Range	737.13; 1,447,538.36	737.13; 396,926.59	5654.86; 1,447,538.36
Time to readmission, days			
Mean (SD)	–	–	8.35 (8.94)
Median (IQR)	–	–	5.00 (0, 15.00)
Range	–	–	0, 30.00
Readmissions, n			
Mean (SD)	–	–	1.78 (1.42)
Median (IQR)	–	–	1.00 (1.00, 2.00)
Range	–	–	1.00, 12.00
Length of hospital stay, days			
Mean (SD)	14.16 (20.26)	8.30 (9.06)	29.49 (30.72)
Median (IQR)	8.00 (4.00, 16.00)	6.00 (4.00, 9.00)	21.00 (12.00, 35.50)
Range	1.00, 376.00	1.00, 189.00	2.00, 376.00

Readmissions occurring within 30 days after discharge were grouped into the previous event. Each event spanned from the admission date of the first decompensation event to the last discharge date.

† Some patients had >1 event.

IQR: Interquartile range; SD: Standard deviation.

The overall mean (SD) cost per event (i.e., hospital stay) was $63,612.09 ($83,281.84; Table 2). The mean (SD) cost per event with readmission within 30 days was $116,424.25 ($120,557.91), and for events without readmission, the mean (SD) cost was $43,404.81 ($50,566.20). In a stratified cost analysis by insurance type, the mean (SD) cost per event was higher for patients with commercial insurance ($82,011.13 [$118,952.84]) versus those with Medicare ($58,984.46 [$70,874.65]).

### Liver transplantation

The mean (SD) total cost of liver transplantation per patient was $328,336.60 ($175,673.49; Table 3). The mean (SD) cost per patient was highest for the hospitalization for transplantation ($226,908.70 [$110,973.59]), followed by costs for post-transplant care and complications ($92,515.85 [$125,261.83]) and the pretransplant evaluation ($11,196.40 [$16,069.89]). In a stratified cost analysis by insurance type, the mean (SD) cost of liver transplantation per patient was higher in patients with commercial insurance ($369,024.39 [$151,850.44]) versus those with Medicare ($271,871.64 [$192,136.46]). The mean (SD) costs for the pretransplant evaluation and hospitalization for transplantation were higher in patients with commercial insurance ($13,190.74 [$19,427.32] and $273,527.62 [$87,037.31], respectively) versus those with Medicare ($8555.95 [$9443.52] and $162,360.33 [$109,098.37], respectively). The mean (SD) cost for post-transplant care and complications was higher in patients with Medicare ($100,955.37 [$140,130.84]) versus those with commercial insurance ($86,005.18 [$113,773.59]).

**Table 3.** Patient-level analysis of liver transplantation cost.

Total cost per patient, $	n = 162^†^
Mean (SD)	328,336.60 (175,673.49)
Median (IQR)	314,975.53 (233,320.86; 387,391.62)
Range	6884.03; 1,099,334.91
Pretransplant evaluation cost per patient, $	n = 162^†^
Mean (SD)	11,196.40 (16,069.89)
Median (IQR)	7127.03 (2703.65; 11,833.71)
Range	292.98; 143,698.87
Hospitalization (for liver transplantation surgery) cost per patient, $	n = 162^†^
Mean (SD)	226,908.70 (110,973.59)
Median (IQR)	273,286.72 (113,589.26; 294,836.70)
Range	937.84; 616,600.58
Post-transplant care and complications cost per patient, $	n = 158
Mean (SD)	92,515.85 (125,261.83)
Median (IQR)	49,045.72 (16,589.85; 116,181.29)
Range	195.22; 757,049.93

† One patient was removed from the analysis due to negative annualized cost.

IQR: Interquartile range; SD: Standard deviation.

## Discussion

This retrospective observational study using a large healthcare claims database demonstrates that hepatic decompensation and liver transplantation present significant economic burden to patients with PBC and healthcare systems, particularly as many patients experience disease progression and its potential complications [2,33]. The mean overall cost of a hospitalization for hepatic decompensation was $63,612.09, which is comparable with inpatient yearly charges for PBC from previously reported studies [20,21]. About a quarter (27.7%) of events resulted in a readmission within 30 days, and the mean cost per event of hepatic decompensation was higher when patients were readmitted within 30 days of discharge compared with events without readmission ($116,424.25 versus $43,404.81). The mean cost of liver transplantation, including pretransplant evaluation, hospitalization for surgery and 1 year of post-transplant care, was $328,336.60, which is similar to previously reported costs [23]. These data highlight the cost burden of PBC disease progression.

This analysis comprised a higher percentage of male patients than other US-based real-world studies of PBC HCRU [20,21,34], with approximately 30% and 50% of patients in the hepatic decompensation and liver transplantation cohorts, respectively, recorded as male. The higher proportion of male patients in this analysis may be explained by prior studies, which have demonstrated that male sex is associated with a higher rate of decompensation [35]. Additionally, approximately 65–70% of patients were White, which is consistent with proportions from previous PBC studies [20,34]. This study’s diverse cohorts are important in understanding the economic burden of PBC and its complications.

This study population had a notable comorbidity burden, as >90% had CCI >1, which is a predictor of 1-year mortality in patients with PBC [21]. Additionally, only approximately half of patients were being treated with UDCA, which is in line with rates from recent studies using longitudinal claims data [22,36] but a lower proportion than that seen in other analyses [3,34,37,38]. While administrative claims data do not contain reasons for prescribing, discontinuation of therapy, or suboptimal UDCA utilization and dosing, this observation may be owing to a lack of historical information because of changes in enrollment, delayed diagnosis, delayed initiation of first-line therapy or previous intolerance to treatment. Further, this study may not fully reflect the longitudinal use of UDCA influenced by adherence, tolerability, lack of historical information because of enrollment changes and potential deductible and copay issues. Nonetheless, the low rate of UDCA use highlights the importance of early diagnosis and timely initiation of first- and second-line therapies to help prevent disease progression and its associated costs, particularly as costs are lower for patients with PBC on treatment [39,40].

### Limitations: missing information

There are several limitations to this analysis. Claims data are collected for billing purposes, and they do not provide potentially important clinical information or context to relevant costs. It is possible that certain data may be missing from the dataset, such as all medications administered during a hospitalization [41], duration of disease and gaps in prescription refills. Some events or comorbidities (assessed based on ICD-9 and ICD-10 codes) may have been omitted due to coding errors. Additionally, the lack of validation against clinical features of PBC (e.g., antimitochondrial antibody positivity, biochemical cholestasis and/or histology), which we cannot assess with claims data, may have potentially affected the rate of UDCA use [4,22,26,42]. Another limitation with claims data is the difficulty in identifying the specific liver-related comorbidity that contributed to hepatic decompensation or liver transplantation.

### Limitations: cost analysis

In order to avoid survival bias, the study required ≥1 day of enrollment after the index date. However, because of this requirement, it is possible that costs were underestimated if patients disenrolled after decompensation or transplantation owing to severe outcomes or terminal events. The cost of death was not estimated. Further, liver transplantation costs may be underestimated as donor costs were not included in the cost analysis. Calculated costs may not reflect the costs negotiated by the insurer, copays, coinsurance or deductibles.

Because indirect costs (e.g., productivity loss, caregiver burden and premature mortality) were not accounted for in the current cost analyses [43], the total cost burden of PBC is likely underestimated. Incorporating both direct and indirect costs may provide further insight to the overall economic burden of PBC. Finally, analyses to identify predictors of cost were beyond the scope of the current study. Additional analytical approaches could provide greater understanding of the cost burden of PBC and represent an important direction for future research.

### Limitations: Optum CDM

Optum CDM includes data from a single national payer and may not reflect the diverse payers across the US. Optum CDM does not include individuals enrolled in Medicaid, those enrolled in Medicare Fee-For-Service, or uninsured individuals. We were unable to assess treatment patterns and HCRU in these populations. As a result, the findings may not be generalizable to these patients, who may be more vulnerable to complications or delays in care [19,44]. As such, costs may be underestimated owing to the exclusion of these potentially high-risk patients. Conversely, it is also possible that costs may be overestimated as patients with commercial insurance may have greater access to healthcare services, resulting in more frequent or higher-cost care. A limited stratified cost analysis demonstrated a higher overall event-level cost of hepatic decompensation and patient-level cost of liver transplantation in patients with commercial insurance compared with Medicare patients. Additional studies are warranted to expand on this analysis and examine the cost burden among patients with various insurance coverages.

In summary, this study provides a comprehensive cost analysis of hospitalization for hepatic decompensation and liver transplantation in patients with PBC. Hepatic decompensation and liver transplantation confer substantial economic burden to patients with PBC and healthcare systems. Additionally, readmissions for hepatic decompensation and complications of transplantation after hospitalization are common, costly, and represent an opportunity for improved care. These data highlight a need for preventing or delaying these disease complications through appropriate management of patients with PBC, which has been shown to improve event-free survival [10–13].

### Summary points

- Primary biliary cholangitis (PBC) confers substantial economic burden to patients and healthcare systems, but the cost of complications of disease progression, specifically hepatic decompensation and liver transplantation, has not yet been quantified.
- This retrospective observational study estimated the real-world cost of hospitalization for hepatic decompensation (including readmissions) and liver transplantation in patients with PBC using a large administrative claims database (Optum’s de-identified Clinformatics^®^ Data Mart Database).
- The mean length of hospital stay for hepatic decompensation was approximately 14 days.
- Among hepatic decompensation events (i.e., hospitalization) with readmissions within 30 days after discharge, the mean time to first readmission was approximately 8 days.
- The mean cost per hepatic decompensation event was $63,612.09.
- The mean cost per hepatic decompensation event with readmission within 30 days was $116,424.25; for events without readmission, the mean cost was $43,404.81.
- The mean total cost of liver transplantation per patient was $328,336.60.
- The mean cost of liver transplantation per patient was highest for the hospitalization ($226,908.70), followed by costs for post-transplant care and complications ($92,515.85) and the pretransplant evaluation ($11,196.40).
- Preventing or delaying PBC-related hepatic decompensation and liver transplantation through appropriate patient management may help to decrease costs for patients and healthcare systems.

## Supplementary Material


