# Supplementary material for: Cost of hepatic decompensation and liver transplantation events in primary biliary cholangitis: a retrospective observational study
Source: J Comp Eff Res. 2026 Feb 16;15(3):e250110. doi: 10.57264/cer-2025-0110 (PMC12976642; doi:10.57264/cer-2025-0110)
Supplement: Supplementary file 1 [file cer-15-250110-s1.docx]

**Supplemental Tables**

**Supplemental Table 1.** Hepatic decompensation-related diagnosis codes and descriptions

| **Code type** | **Diagnosis code** | **Description** |
| --- | --- | --- |
| ICD-10-CM | K70.11 | Alcoholic hepatitis with ascites |
| ICD-10-CM | K70.31 | Alcoholic cirrhosis of liver with ascites |
| ICD-10-CM | K71.51 | Toxic liver disease with chronic active hepatitis with ascites |
| ICD-10-CM | R18.0 | Malignant ascites |
| ICD-10-CM | R18.8 | Other ascites |
| ICD-9-CM | 568.82 | Peritoneal effusion (chronic) |
| ICD-9-CM | 789.51 | Malignant ascites |
| ICD-9-CM | 789.59 | Other ascites |
| ICD-10-CM | K65.2 | Spontaneous bacterial peritonitis |
| ICD-9-CM | 567.23 | Spontaneous bacterial peritonitis |
| ICD-10-CM | R17 | Unspecified jaundice |
| ICD-9-CM | 782.4 | Jaundice, unspecified, not of newborn |
| ICD-10-CM | I85.01 | Esophageal varices with bleeding |
| ICD-10-CM | I85.11 | Secondary esophageal varices with bleeding |
| ICD-9-CM | 456.0 | Esophageal varices with bleeding |
| ICD-9-CM | 456.20 | Esophageal varices in diseases classified elsewhere, with bleeding |
| ICD-9-CM | 572.2 | Hepatic encephalopathy |
| ICD-10-CM | K72.90 | Hepatic failure, unspecified without coma |
| ICD-10-CM | K72.91 | Hepatic failure, unspecified with coma |
| ICD-10-CM | K70.41 | Alcoholic hepatic failure with coma |
| ICD-10-CM | K71.11 | Toxic liver disease with hepatic necrosis, with coma |
| ICD-10-CM | K72.01 | Acute and subacute hepatic failure with coma |
| ICD-10-CM | K72.11 | Chronic hepatic failure with coma |
| ICD-10-CM | K76.82 | Hepatic encephalopathy |
| ICD-10-CM | K76.6 | Portal hypertension |
| ICD-9-CM | 572.3 | Portal hypertension |

Abbreviations: CM, Clinical Modification; ICD, International Classification of Diseases.

**Supplemental Table 2.** Hepatic decompensation-related procedure codes and descriptions

| **Code type** | **Diagnosis code** | **Description** |
| --- | --- | --- |
| CPT | 49082 | Abdominal paracentesis (diagnostic or therapeutic); without imaging guidance |
| CPT | 49083 | Abdominal paracentesis (diagnostic or therapeutic); with imaging guidance |
| ICD-10-PCS | 0W9G00Z | Drainage of Peritoneal Cavity with Drainage Device, Open Approach |
| ICD-10-PCS | 0W9G0ZX | Drainage of Peritoneal Cavity, Open Approach, Diagnostic |
| ICD-10-PCS | 0W9G0ZZ | Drainage of Peritoneal Cavity, Open Approach |
| ICD-10-PCS | 0W9G30Z | Drainage of Peritoneal Cavity with Drainage Device, Percutaneous Approach |
| ICD-10-PCS | 0W9G3ZX | Drainage of Peritoneal Cavity, Percutaneous Approach, Diagnostic |
| ICD-10-PCS | 0W9G3ZZ | Drainage of Peritoneal Cavity, Percutaneous Approach |
| ICD-10-PCS | 0W9G40Z | Drainage of Peritoneal Cavity with Drainage Device, Percutaneous Endoscopic Approach |
| ICD-10-PCS | 0W9G4ZX | Drainage of Peritoneal Cavity, Percutaneous Endoscopic Approach, Diagnostic |
| ICD-10-PCS | 0W9G4ZZ | Drainage of Peritoneal Cavity, Percutaneous Endoscopic Approach |
| ICD-9-PCS | 54.91 | Percutaneous abdominal drainage |
| CPT | 37182 | Insertion of transvenous intrahepatic portosystemic shunt(s) (TIPS) (includes venous access, hepatic and portal vein catheterization, portography with hemodynamic evaluation, intrahepatic tract formation/dilatation, stent placement and all associated imaging guidance and documentation) |
| CPT | 37183 | Revision of transvenous intrahepatic portosystemic shunt(s) (TIPS) (includes venous access, hepatic and portal vein catheterization, portography with hemodynamic evaluation, intrahepatic tract recannulization/dilatation, stent placement and all associated imaging guidance and documentation) |
| ICD-10-PCS | 06183J4 | Bypass Portal Vein to Hepatic Vein with Synthetic Substitute, Percutaneous Approach |
| ICD-10-PCS | 06184J4 | Bypass Portal Vein to Hepatic Vein with Synthetic Substitute, Percutaneous Endoscopic Approach |
| ICD-10-PCS | 06H43DZ | Insertion of Intraluminal Device into Hepatic Vein, Percutaneous Approach |
| ICD-10-PCS | 06H44DZ | Insertion of Intraluminal Device into Hepatic Vein, Percutaneous Endoscopic Approach |
| ICD-10-PCS | 06H83DZ | Insertion of Intraluminal Device into Portal Vein, Percutaneous Approach |
| ICD-10-PCS | 06H84DZ | Insertion of Intraluminal Device into Portal Vein, Percutaneous Endoscopic Approach |
| ICD-9-PCS | 39.1 | Intra-abdominal venous shunt |
| CPT | 37181 | Venous anastomosis, open; splenorenal, distal (selective decompression of esophagogastric varices, any technique) |
| CPT | 43204 | Esophagoscopy, flexible, transoral; with injection sclerosis of esophageal varices |
| CPT | 43205 | Esophagoscopy, flexible, transoral; with band ligation of esophageal varices |
| CPT | 43243 | Esophagogastroduodenoscopy, flexible, transoral; with injection sclerosis of esophageal/gastric varices |
| CPT | 43244 | Esophagogastroduodenoscopy, flexible, transoral; with band ligation of esophageal/gastric varices |
| CPT | 43400 | Ligation, direct, esophageal varices |
| CPT | 43460 | Esophagogastric tamponade, with balloon (Sengstaken type) |
| ICD-10-PCS | 0611079 | Bypass Splenic Vein to Right Renal Vein with Autologous Tissue Substitute, Open Approach |
| ICD-10-PCS | 0611099 | Bypass Splenic Vein to Right Renal Vein with Autologous Venous Tissue, Open Approach |
| ICD-10-PCS | 0611479 | Bypass Splenic Vein to Right Renal Vein with Autologous Tissue Substitute, Percutaneous Endoscopic Approach |
| ICD-10-PCS | 0611499 | Bypass Splenic Vein to Right Renal Vein with Autologous Venous Tissue, Percutaneous Endoscopic Approach |
| ICD-10-PCS | 061107B | Bypass Splenic Vein to Left Renal Vein with Autologous Tissue Substitute, Open Approach |
| ICD-10-PCS | 061109B | Bypass Splenic Vein to Left Renal Vein with Autologous Venous Tissue, Open Approach |
| ICD-10-PCS | 06110A9 | Bypass Splenic Vein to Right Renal Vein with Autologous Arterial Tissue, Open Approach |
| ICD-10-PCS | 06110AB | Bypass Splenic Vein to Left Renal Vein with Autologous Arterial Tissue, Open Approach |
| ICD-10-PCS | 06110J9 | Bypass Splenic Vein to Right Renal Vein with Synthetic Substitute, Open Approach |
| ICD-10-PCS | 06110JB | Bypass Splenic Vein to Left Renal Vein with Synthetic Substitute, Open Approach |
| ICD-10-PCS | 06110K9 | Bypass Splenic Vein to Right Renal Vein with Nonautologous Tissue Substitute, Open Approach |
| ICD-10-PCS | 06110KB | Bypass Splenic Vein to Left Renal Vein with Nonautologous Tissue Substitute, Open Approach |
| ICD-10-PCS | 06110Z9 | Bypass Splenic Vein to Right Renal Vein, Open Approach |
| ICD-10-PCS | 06110ZB | Bypass Splenic Vein to Left Renal Vein, Open Approach |
| ICD-10-PCS | 061147B | Bypass Splenic Vein to Left Renal Vein with Autologous Tissue Substitute, Percutaneous Endoscopic Approach |
| ICD-10-PCS | 061149B | Bypass Splenic Vein to Left Renal Vein with Autologous Venous Tissue, Percutaneous Endoscopic Approach |
| ICD-10-PCS | 06114A9 | Bypass Splenic Vein to Right Renal Vein with Autologous Arterial Tissue, Percutaneous Endoscopic Approach |
| ICD-10-PCS | 06114AB | Bypass Splenic Vein to Left Renal Vein with Autologous Arterial Tissue, Percutaneous Endoscopic Approach |
| ICD-10-PCS | 06114J9 | Bypass Splenic Vein to Right Renal Vein with Synthetic Substitute, Percutaneous Endoscopic Approach |
| ICD-10-PCS | 06114JB | Bypass Splenic Vein to Left Renal Vein with Synthetic Substitute, Percutaneous Endoscopic Approach |
| ICD-10-PCS | 06114K9 | Bypass Splenic Vein to Right Renal Vein with Nonautologous Tissue Substitute, Percutaneous Endoscopic Approach |
| ICD-10-PCS | 06114KB | Bypass Splenic Vein to Left Renal Vein with Nonautologous Tissue Substitute, Percutaneous Endoscopic Approach |
| ICD-10-PCS | 06114Z9 | Bypass Splenic Vein to Right Renal Vein, Percutaneous Endoscopic Approach |
| ICD-10-PCS | 06114ZB | Bypass Splenic Vein to Left Renal Vein, Percutaneous Endoscopic Approach |
| ICD-10-PCS | 06L30CZ | Occlusion of Esophageal Vein with Extraluminal Device, Open Approach |
| ICD-10-PCS | 06L30DZ | Occlusion of Esophageal Vein with Intraluminal Device, Open Approach |
| ICD-10-PCS | 06L30ZZ | Occlusion of Esophageal Vein, Open Approach |
| ICD-10-PCS | 06L33CZ | Occlusion of Esophageal Vein with Extraluminal Device, Percutaneous Approach |
| ICD-10-PCS | 06L33DZ | Occlusion of Esophageal Vein with Intraluminal Device, Percutaneous Approach |
| ICD-10-PCS | 06L33ZZ | Occlusion of Esophageal Vein, Percutaneous Approach |
| ICD-10-PCS | 06L34CZ | Occlusion of Esophageal Vein with Extraluminal Device, Percutaneous Endoscopic Approach |
| ICD-10-PCS | 06L34DZ | Occlusion of Esophageal Vein with Intraluminal Device, Percutaneous Endoscopic Approach |
| ICD-10-PCS | 06L34ZZ | Occlusion of Esophageal Vein, Percutaneous Endoscopic Approach |
| ICD-10-PCS | 06L37CZ | Occlusion of Esophageal Vein with Extraluminal Device, Via Natural or Artificial Opening |
| ICD-10-PCS | 06L37DZ | Occlusion of Esophageal Vein with Intraluminal Device, Via Natural or Artificial Opening |
| ICD-10-PCS | 06L37ZZ | Occlusion of Esophageal Vein, Via Natural or Artificial Opening |
| ICD-10-PCS | 06L38CZ | Occlusion of Esophageal Vein with Extraluminal Device, Via Natural or Artificial Opening Endoscopic |
| ICD-10-PCS | 06L38DZ | Occlusion of Esophageal Vein with Intraluminal Device, Via Natural or Artificial Opening Endoscopic |
| ICD-10-PCS | 06L38ZZ | Occlusion of Esophageal Vein, Via Natural or Artificial Opening Endoscopic |
| ICD-10-PCS | 06N20ZZ | Release Gastric Vein, Open Approach |
| ICD-10-PCS | 06N23ZZ | Release Gastric Vein, Percutaneous Approach |
| ICD-10-PCS | 06N24ZZ | Release Gastric Vein, Percutaneous Endoscopic Approach |
| ICD-10-PCS | 06N30ZZ | Release Esophageal Vein, Open Approach |
| ICD-10-PCS | 06N33ZZ | Release Esophageal Vein, Percutaneous Approach |
| ICD-10-PCS | 06N34ZZ | Release Esophageal Vein, Percutaneous Endoscopic Approach |
| ICD-10-PCS | 0DL57DZ | Occlusion of Esophagus with Intraluminal Device, Via Natural or Artificial Opening |
| ICD-9-PCS | 42.33 | Endoscopic excision or destruction of lesion or tissue of esophagus |
| ICD-9-PCS | 42.91 | Ligation of esophageal varices |
| ICD-9-PCS | 43.41 | Endoscopic excision or destruction of lesion or tissue of stomach |
| ICD-9-PCS | 96.06 | Insertion of Sengstaken tube |

Abbreviations: CPT, Current Procedural Terminology; ICD, International Classification of Diseases; PCS, Procedure Coding System.

**Supplemental Table 3.** Health Care Provider Taxonomy codes for post-discharge^a^ and post-transplant^b^ specialist visits

| **Code** | **Name** |
| --- | --- |
| 207RG0100X | Gastroenterology |
| 2080P0206X | Pediatric gastroenterology |
| 207RI0008X | Hepatology |
| 207RT0003X | Transplant hepatology |
| 2080T0004X | Pediatric transplant hepatology |

^a^Specialist visits during the 30-day post-discharge period were included in the cost of the hepatic decompensation event.

^b^Specialist visits during the 1 year following liver transplantation were included in the cost of post-transplant care and complications.

**Supplemental Table 4.** Clinical evaluations, laboratory tests, procedures, treatments, and encounters included in the pre-transplant evaluation^a^

| **Evaluation** | **ICD-10-CM diagnosis codes** | **ICD-9-CM diagnosis codes** | **Health Care Provider Taxonomy codes** | **Laboratory tests, procedures, treatments, and encounters** |
| --- | --- | --- | --- | --- |
| **Gastroenterologist/hepatologist encounter** | N/A | N/A | - 207RG0100X (gastroenterology) - 2080P0206X (pediatric gastroenterology) - 207RI0008X (hepatology) - 207RT0003X (transplant hepatology) - 2080T0004X (pediatric transplant hepatology) | N/A |
| **General health assessment** | - Z01.4 (Encounter for gynecological examination) - Z01.81 (Encounter for preprocedural examinations) | - V72.3 (Gynecological examination) - V72.63 (Pre-procedural laboratory examination) - V72.81 (Pre-operative cardiovascular examination) - V72.82 (Pre-operative respiratory examination) - V72.83 (Other specified pre-operative examination) - V72.84 (Pre-operative examination, unspecified) | N/A | - Chest X-ray - Mammogram - Pap smear - Colonoscopy |
| **Dental assessment** | - Z01.2 (Encounter for dental examination and cleaning) - Z13.84 (Encounter for screening for dental disorders) | - V72.2 (Dental examination) | N/A |  |
| **Psychiatry, psychology, or mental health professional consultation** | - Z13.3 (Encounter for screening examination for mental health and behavioral disorders) | N/A | - 2084A0401X (addiction medicine [psychiatry and neurology] physician - 2084B0002X (obesity medicine [psychiatry and neurology] physician) - 2084F0202X (forensic psychiatry physician) - 2084H0002X (hospice and palliative medicine [psychiatry and neurology] physician - 2084N0008X (neuromuscular medicine [psychiatry and neurology] physician - 2084P0015X (psychosomatic medicine physician) - 2084P0301X (brain injury medicine [psychiatry and neurology] physician - 2084P0800X (psychiatry physician) - 2084P0802X (addiction psychiatry physician) - 2084P0804X (child and adolescent psychiatry physician) - 2084P0805X (geriatric psychiatry physician) - 2084P2900X (pain medicine [psychiatry and neurology] physician - 2084S0012X (sleep medicine [psychiatry and neurology] physician - 103TA0400X (addiction [substance use disorder] psychologist - 103TA0700X (adult development and aging psychologist) - 103TB0200X (cognitive and behavioral psychologist) - 103TC0700X (clinical psychologist) - 103TC1900X (counseling psychologist) - 103TC2200X (clinical child and adolescent psychologist) - 103TF0000X (family psychologist) - 103TH0004X (health psychologist) - 103TH0100X (health service psychologist) - 103TP0016X (prescribing [medical] psychologist) - 103TP2701X (group psychotherapy psychologist) | - Any outpatient encounter with psychiatry or psychology |
| **Nutritional evaluation** | - Z13.21 (Encounter for screening for nutritional disorder) - Z13.220 (Encounter for screening for lipoid disorders) | - V77.2 (Screening for malnutrition) - V77.9 (Special screening for other and unspecified endocrine nutritional metabolic and immunity disorders) | N/A | - Medical nutrition therapy |
| **Cardiac evaluation** | N/A | N/A | - 207RA0001X (advanced heart failure and transplant cardiology physician) - 207RA0002X (adult congenital heart disease physician) - 207RC0000X (cardiovascular disease) - 207RC0001X (clinical care electrophysiology) - 207RI0011X (interventional cardiology physician) - 207UN0901X (nuclear cardiology physician) - 2080P0202X (pediatric cardiology physician) | - Any outpatient encounter with cardiology - Echocardiography - Cardiology stress testing |
| **Laboratory testing** | - Z01.83 (Encounter for blood typing) - Z02.83 (Encounter for blood-alcohol and blood-drug test) | - V72.86 (Encounter for blood typing) - V70.4 (Examination for medicolegal reasons) | N/A | - ABO-Rh blood typing - Liver panel (ALT, AST, ALP, bilirubin [direct and total]) - INR - CBC with differential - Creatinine clearance - Serum alpha-fetoprotein - Serum calcium - Serum vitamin D - Serum phosphatidyl ethanol - Urinalysis - Urine drug screen |
| **Infection screening** | - Z11.0 (Encounter for screening for intestinal infectious diseases) - Z11.1 (Encounter for screening for respiratory tuberculosis) - Z11.2 (Encounter for screening for other bacterial diseases) - Z11.3 (Encounter for screening for infections with a predominantly sexual mode of transmission) - Z11.4 (Encounter for screening for human immunodeficiency virus [HIV]) - Z11.5 (Encounter for screening for other viral diseases) - Z11.7 (Encounter for testing for latent tuberculosis infection) - Z11.8 (Encounter for screening for other infectious and parasitic diseases) - Z11.9 (Encounter for screening for infectious and parasitic diseases, unspecified) | - V73 (Special screening examination for viral and chlamydial diseases) - V74 (Special screening examination for bacterial and spirochetal diseases) - V75.0 (Screening examination for rickettsial diseases) - V75.1 (Screening examination for malaria) - V75.2 (Screening examination for leishmaniasis) - V75.3 (Screening examination for trypanosomiasis) - V75.4 (Screening examination for mycotic infections) - V75.5 (Screening examination for schistosomiasis) - V75.6 (Screening examination for filariasis) - V75.9 (Screening examination for unspecified infectious disease) | N/A | Serology for:   - CMV - EBV - Varicella - HIV - Hepatitis A - Hepatitis B - Hepatitis C - RPR - TB |
| **Hepatic imaging** | N/A | N/A | N/A | - Doppler ultrasound of the liver - Triple-phase abdominal CT scan - Gadolinium-enhanced abdominal MRI |

Abbreviations: ALP, alkaline phosphatase; ALT, alanine aminotransferase; AST, aspartate aminotransferase; CBC, complete blood count; CM, clinical modification; CMV, cytomegalovirus; CT, computed tomography; EBV, Epstein-Barr virus; ICD, International Classification of Diseases; INR, international normalized ratio; MRI, magnetic resonance imaging; RPR, rapid plasma reagin; TB, tuberculosis.

Note: Select information derived from Martin P, DiMartini A, Feng S, Brown R Jr, Fallon M. Evaluation for liver transplantation in adults: 2013 practice guideline by the American Association for the Study of Liver Diseases and the American Society of Transplantation. *Hepatology*. 2014;59(3):1144-1165.

^a^Within 6 months before liver transplantation.

**Supplemental Table 5.** Current Procedural Terminology codes and descriptions for liver transplantation procedures

| **CPT code** | **Description** |
| --- | --- |
| 47133 | Donor hepatectomy (including cold preservation), from cadaver donor |
| 47135 | Liver allotransplantation; orthotopic, partial or whole, from cadaver or living donor, any age |
| 47140 | Donor hepatectomy (including cold preservation), from living donor; left lateral segment only (segments II and III) |
| 47141 | Donor hepatectomy (including cold preservation), from living donor; total left lobectomy (segments II, III and IV) |
| 47142 | Donor hepatectomy (including cold preservation), from living donor; total right lobectomy (segments V, VI, VII and VIII) |
| 47143 | Backbench standard preparation of cadaver donor whole liver graft prior to allotransplantation, including cholecystectomy |
| 47144 | Backbench standard preparation of cadaver donor whole liver graft prior to allotransplantation, including cholecystectomy |
| 47145 | Backbench standard preparation of cadaver donor whole liver graft prior to allotransplantation, including cholecystectomy, if necessary, and dissection and removal of surrounding soft tissues to prepare the vena cava, portal vein, hepatic artery, and common bile duct for implantation |
| 47146 | Backbench reconstruction of cadaver or living donor liver graft prior to allotransplantation; venous anastomosis |
| 47147 | Backbench reconstruction of cadaver or living donor liver graft prior to allotransplantation; arterial anastomosis |

Abbreviation: CPT, Current Procedural Terminology.
